# Total mRNA Quantification in Single Cells: Sarcoma Cell Heterogeneity

**DOI:** 10.3390/cells9030759

**Published:** 2020-03-19

**Authors:** Emma Jonasson, Lisa Andersson, Soheila Dolatabadi, Salim Ghannoum, Pierre Åman, Anders Ståhlberg

**Affiliations:** 1 Sahlgrenska Cancer Center, Department of Laboratory Medicine, Institute of Biomedicine, Sahlgrenska Academy at University of Gothenburg, SE-405 30 Gothenburg, Sweden; emma.jonasson@gu.se (E.J.); lisa.andersson.3@gu.se (L.A.); soheila.dolatabadi@gu.se (S.D.); salim.ghannoum@medisin.uio.no (S.G.); pierre.aman@gu.se (P.Å.); 2 Department of Clinical Genetics and Genomics, Sahlgrenska University Hospital, SE-405 30 Gothenburg, Sweden; 3 Wallenberg Centre for Molecular and Translational Medicine, University of Gothenburg, SE-405 30 Gothenburg, Sweden

**Keywords:** cell heterogeneity, sarcoma, single-cell analysis, total mRNA level, transcriptome size

## Abstract

Single-cell analysis enables detailed molecular characterization of cells in relation to cell type, genotype, cell state, temporal variations, and microenvironment. These studies often include the analysis of individual genes and networks of genes. The total amount of RNA also varies between cells due to important factors, such as cell type, cell size, and cell cycle state. However, there is a lack of simple and sensitive methods to quantify the total amount of RNA, especially mRNA. Here, we developed a method to quantify total mRNA levels in single cells based on global reverse transcription followed by quantitative PCR. Standard curve analyses of diluted RNA and sorted cells showed a wide dynamic range, high reproducibility, and excellent sensitivity. Single-cell analysis of three sarcoma cell lines and human fibroblasts revealed cell type variations, a lognormal distribution of total mRNA levels, and up to an eight-fold difference in total mRNA levels among the cells. The approach can easily be combined with targeted or global gene expression profiling, providing new means to study cell heterogeneity at an individual gene level and at a global level. This method can be used to investigate the biological importance of variations in the total amount of mRNA in healthy as well as pathological conditions.

## 1. Introduction

Gene expression profiling is widely used in both research and medicine for the characterization of different biological and pathological conditions. Normally, these experiments are performed on bulk samples that include populations of cells. However, it is well-known that there exist large variations in gene expression levels between individual cells caused by cell type, cell state, genotype, temporal variations in gene expression, and microenvironment [1]. Gene expression analysis at the cell population level cannot reveal any information about this cellular heterogeneity. Single-cell gene expression profiling has emerged as a tool to resolve this issue, and several technologies are today available, from targeted quantitative PCR (qPCR) [2] to a wide range of high-throughput RNA sequencing protocols [3]. Single-cell gene expression profiling has been applied to a variety of different biological and clinical applications, including cell type characterization [4], hierarchical organization of hematopoietic progenitors [5], the immune response to bacterial infection [6], therapy resistance in cancer [7], and mapping of intratumoral heterogeneity [8]. Another issue with traditional bulk gene expression profiling is that data are compared between samples after global normalization or after normalization to specific reference genes. These normalization strategies assume that the total amounts of transcripts should be equal among the samples compared. However, this assumption is not always valid since differences in the total amounts of both mRNA and other RNAs have been associated with several biological factors [9]. These include cell type [10], cell size [11], cell cycle state [12], and aging [13], and it has also been shown that some proteins, for example, the oncogenic transcription factor c-Myc [14,15] and methyl CpG binding protein 2 (MECP2) [16], affect the transcriptome globally in certain cell types. These differences in total RNA levels will be obscured using traditional normalization methods, resulting in quantitative biases [17]. At the single-cell level, the transcript levels of individual genes, as well as the total mRNA level, can be analyzed per cell and thereby be compared more directly [18,19]. The biological importance of variations in the total amount of mRNA and other RNAs is partly unknown, and more studies are needed. To achieve this, there is a need for easy and accurate methods to measure the total RNA amount, especially the total mRNA amount, in single cells.

Here, we developed a fast, easy, and flexible method to measure the total mRNA level in single cells. The approach reverse-transcribes polyadenylated RNA, followed by global amplification of the resulting pool of complementary DNA. The method does not require sequencing, but can easily be combined with both targeted qPCR and global RNA sequencing for additional cell analysis. We applied the method to three different types of sarcomas, including myxoid liposarcoma (MLS), fibrosarcoma, and Ewing sarcoma (EWS), as well as to short-term cultured fibroblasts.

## 2. Materials and Methods

### 2.1. Cell Culture

The MLS cell line 2645-94 [20] and the fibrosarcoma cell line HT1080 [21] were cultured in RPMI1640 GlutaMAX medium supplied with 5% fetal bovine serum, 100 U/mL penicillin, and 100 μg/mL streptomycin (all Gibco, Thermo Fisher Scientific, Waltham, MA, USA). The EWS cell line TC-71 [22] was cultured in Iscove’s Modified Dulbecco’s medium (Gibco, Thermo Fisher Scientific, Waltham, MA, USA) supplemented with 10% fetal bovine serum, 100 U/mL penicillin, and 100 μg/mL streptomycin. Normal skin fibroblasts F470 were cultured in RPMI1640 GlutaMAX medium supplied with 10% fetal bovine serum, 100 U/mL penicillin, and 100 μg/mL streptomycin. Cells were passaged using 0.25% trypsin (Gibco, Thermo Fisher Scientific, Waltham, MA, USA) supplemented with 0.5 mM EDTA (Invitrogen, Thermo Fisher Scientific, Waltham, MA, USA) and maintained at 37 °C in 5% CO_2_. All analyzed cells were non-synchronized and in a non-confluent state.

### 2.2. Total RNA Extraction

Cells cultured in monolayers were washed once with Dulbecco’s phosphate-buffered saline (Gibco, Thermo Fisher Scientific, Waltham, MA, USA), and thereafter directly lysed by adding QIAzol Lysis Reagent (Qiagen, Hilden, Germany). The lysate was collected with a cell scraper (Falcon, VWR, Radnor, PA, USA), transferred to a microcentrifuge tube, vortexed, and immediately frozen on dry ice. The homogenized cell lysates were stored at -80 °C until RNA isolation. RNA was extracted from cells using the miRNeasy micro kit (Qiagen, Hilden, Germany), according to the manufacturer’s instructions, including DNase treatment. The purification procedure was performed manually or automated on a QIAcube (Qiagen, Hilden, Germany). Samples were eluted in 14 µL RNase/DNase-free water (Invitrogen, Thermo Fisher Scientific, Waltham, MA, USA), and their concentrations were quantified with Qubit fluorometer (Invitrogen, Thermo Fisher Scientific, Waltham, MA, USA). Isolated RNA was stored at −80 °C. RNA dilutions were performed with a single-cell lysis buffer containing 1 µg/µL bovine serum albumin supplied in 2.5% glycerol (Thermo Scientific, Thermo Fisher Scientific, Waltham, MA, USA) and 0.2% Triton X-100 (Sigma-Aldrich, St. Louis, MO, USA). RNA integrity was assessed on a Fragment Analyzer using the DNF-471 RNA kit (both Agilent Technologies, Santa Clara, CA, USA), according to manufacturer’s instructions, and data were processed with the PROSize3 data analysis software (Agilent Technologies, Santa Clara, CA, USA).

### 2.3. Single-Cell Collection

Cells were detached using 0.25% trypsin supplemented with 0.5 mM EDTA, and trypsin was inactivated with complete media. Cells were resuspended in Hank’s Balanced Salt Solution (Gibco, Thermo Fisher Scientific, Waltham, MA, USA) and stained with 0.9 µM propidium iodide (Sigma-Aldrich, St. Louis, MO, USA) for 5 min at room temperature, followed by centrifugation and resuspension in Hank’s Balanced Salt Solution. A single-cell suspension was generated by passing the cells through a cell strainer with a pore size of 70 μm (Corning Life Sciences, Amsterdam, The Netherlands).

Fluorescence-activated cell sorting was performed using a BD FACSAria II or a BD FACSAria Fusion instrument and the FACSDiva software (all BD Biosciences, San Jose, CA, USA). Single cells were sorted into 96-well PCR plates (Applied Biosystems, Thermo Fisher Scientific, Waltham, MA, USA) with 5 µL lysis buffer containing 1 µg/µL bovine serum albumin supplied in 2.5% glycerol and 0.2% Triton X-100. Viable cells were selected based on dye exclusion of propidium iodide. Negative controls were wells without any sorted cells. After sorting, plates were immediately frozen on dry ice and stored at −80 °C until reverse transcription.

### 2.4. Total Polyadenylated RNA Analysis

The total amount of polyadenylated transcripts was analyzed by reverse transcription of full-length polyadenylated RNA followed by cDNA quantification using qPCR. The approach was based on the Smart-seq2 protocol [23].

Reverse transcription was performed using either extracted total RNA diluted in 5 µL lysis buffer containing 1 µg/µL bovine serum albumin supplied in 2.5% glycerol and 0.2% Triton X-100, or direct-lysed single cells. First, 1 µM biotinylated adapter sequence-containing oligo-dT30VN (5′-Biotin-AAGCAGTGGTATCAACGCAGAGTACT30VN-3′; Sigma-Aldrich, St. Louis, MO, USA, or IDT technologies, Coralville, IA, USA) and 1 mM dNTP (Sigma-Aldrich, St. Louis, MO, USA) were added to the sample followed by incubation at 72 °C for 3 min and cooling to 4 °C. Next, 1x first-strand buffer (50 mM Tris-HCl pH 8.3, 75 mM KCl, and 3 mM MgCl_2_), 5 mM dithiothreitol (both Invitrogen, Thermo Fisher Scientific, Waltham, MA, USA), 10 mM MgCl_2_ (Ambion, Thermo Fisher Scientific, Waltham, MA, USA), 1 M betaine (Sigma-Aldrich, St. Louis, MO, USA), 0.6 µM biotinylated adapter sequence-containing template-switching oligonucleotide (5′-Biotin-AAGCAGTGGTATCAACGCAGAGTACATrGrG+G-3′ with rG = riboguanosine and +G = locked nucleic acid-modified guanosine, Eurogentec, Liège, Belgien), 15 U RNaseOUT, and 150 U SuperScript II (both Invitrogen, Thermo Fisher Scientific, Waltham, MA, USA) were added to a final volume of 15 µL, and reverse transcription was performed in a T100 instrument (Bio-Rad, Hercules, CA, USA) at 42 °C for 90 min and 70 °C for 15 min. Final reaction concentrations are indicated. Complementary DNA was stored at −20 °C.

Complementary DNA quantification was performed in a 30 µL reaction containing 1x KAPA Hifi HotStart Ready Mix (KAPA Biosystems, Wilmington, MA, USA), 0.1 µM adapter primer (5′-AAGCAGTGGTATCAACGCAGAGT-3′; Sigma-Aldrich, St. Louis, MO, USA, or IDT technologies, Coralville, IA, USA), 0.5x SYBR Green I (Invitrogen, Thermo Fisher Scientific, Waltham, MA, USA), and 4.5 µL cDNA using a CFX384 Touch Real-Time PCR Detection System (Bio-Rad, Hercules, CA, USA). The temperature profile used was: 98 °C for 3 min, followed by 35 cycles of amplification at 98 °C for 20 s, 67 °C for 15 s, and 72 °C for 6 min with a final additional incubation at 72 °C for 5 min and a melting curve analysis, ranging from 65 °C to 95 °C with an increase of 0.1 °C per second.

Cycles of quantification values were determined by threshold using the CFX Manager Software version 3.1 (Bio-Rad, Hercules, CA, USA). PCR efficiencies were determined based on the linear regression of standard curves. The PCR efficiencies calculated from FACS-sorted cells were used to convert the cycle of quantification values to relative quantities for single-cell data, with a value equal to one for the lowest expression value. Relative quantities were log-transformed. To compensate for interplate variation, an RNA interplate calibrator was used as described [24]. Statistical analysis was performed using Prism version 8.2.1 (GraphPad Software Inc., La Jolla, CA, USA). For library quality assessment, preamplification was performed for a limited number of cycles (24 cycles for single cells and 18 cycles for 128 cells, respectively) using the cDNA quantification protocol, omitting melting curve analysis, as cycling beyond the exponential phase can introduce biases for downstream analyses [25]. As a control, the same samples were analyzed without adding SYBR Green I to the reaction. Preamplified samples were purified using Agencourt AMPure XP beads (BD Biosciences, San Jose, CA, USA) with a beads-to-sample ratio of 0.8. Beads were mixed with samples through pipetting, followed by incubation for 5 min at room temperature and 5 min on a magnet (DynaMag, Thermo Fisher Scientific, Waltham, MA, USA). After discarding the supernatant, the DNA-bound beads were washed twice with 200 µL 80% ethanol (Solveco, Rosersberg Sweden). The beads were left to dry, and the purified DNA was eluted by mixing the samples with 17.5 µL RNase/DNase-free water, followed by incubation for 2 min at room temperature and 2 min on a magnet, before 15 µL of each sample was retrieved.

Preamplified cDNA integrity was assessed on a Fragment Analyzer using the DNF-474 High Sensitivity NGS kit (Agilent technologies, Santa Clara, CA, USA). Analyses were performed, according to the manufacturer’s instructions, and data were analyzed with PROSize3 data analysis software.

## 3. Results

### 3.1. Development of a Method to Quantify the Polyadenylated Transcriptome of Single Cells

**Figure 1:**
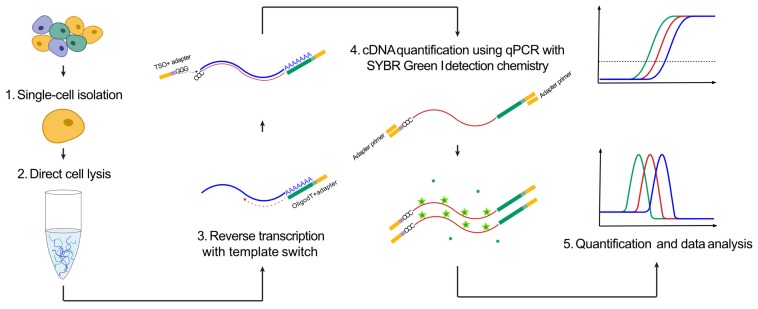
Total polyadenylated RNA analysis. The experimental approach to quantify polyadenylated RNA.

To quantify the amount of polyadenylated RNA in individual cells, we developed a fast and simple approach based on full-length reverse transcription of RNA, followed by qPCR with SYBR Green I detection chemistry. The strategy was based on Smart-seq2 [23] that enables full-length reverse transcription of polyadenylated RNA using a template-switching oligo, generating cDNA with a common adapter in each sequence end (Figure 1). The cDNA is then preamplified by PCR using a single primer. Here, we applied the same reverse transcription step, but the amount of generated cDNA was quantified using qPCR with SYBR Green I detection chemistry. To assess the formation of specific PCR products, the qPCR was followed by a melting curve analysis.

**Figure 2:**
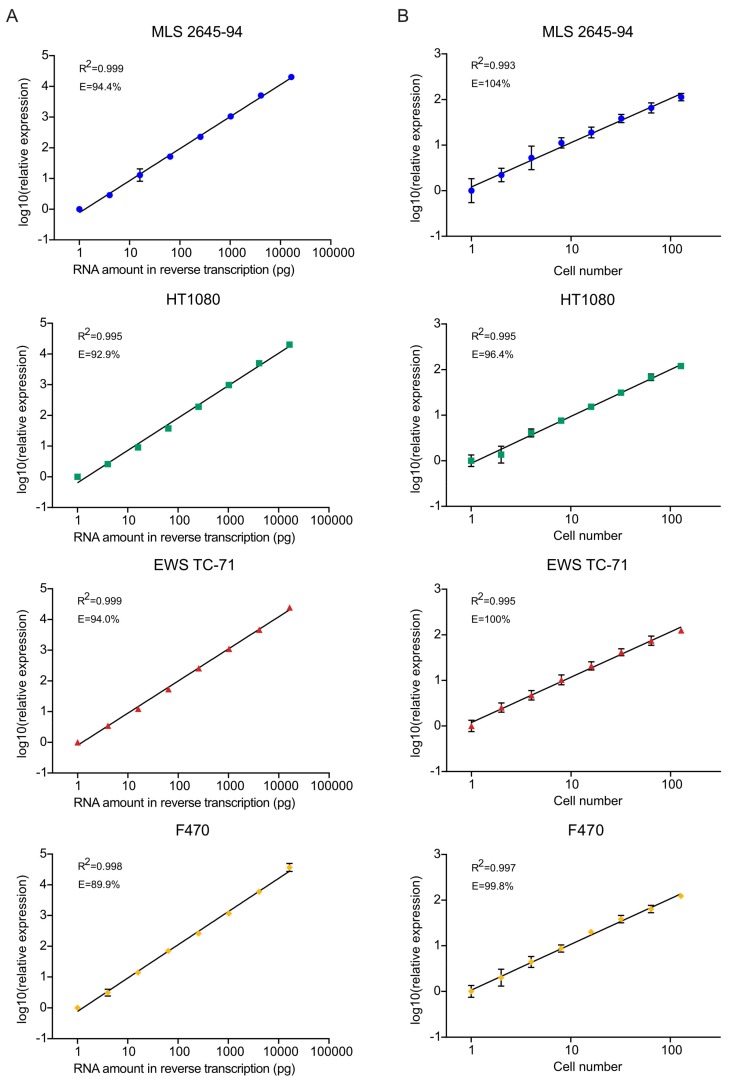
Total polyadenylated RNA analysis. (**A**) Total polyadenylated RNA analysis of different amounts of total RNA extracted from myxoid liposarcoma (MLS) 2645-94, HT1080, Ewing sarcoma (EWS) TC-71, and F470. Standard curves ranged from 16.4 ng to 1 pg with dilution steps of four. The relationship between relative quantity and RNA amount was tested with linear regression. Mean ± SD is shown, *n* = 3–5. PCR efficiencies (E) and R^2^ values are indicated. (**B**) Total polyadenylated RNA analysis of a different number of cells sorted from MLS 2645-94, HT1080, EWS TC-71, and F470. Standard curves ranged from 128 cells to single cells in steps of two. The relationship between relative quantity and cell number was tested with linear regression. Mean ± SD is shown, *n* = 4–7 (>1 cell), *n* = 6–14 (one cell). PCR efficiencies (E) and R^2^ values are indicated.

To determine the efficiency, reproducibility, and dynamic range of the approach, we performed standard curves of extracted total RNA from MLS (MLS 2645-94), fibrosarcoma (HT1080), EWS (EWS TC-71), and skin fibroblasts (F470), ranging from 16.4 ng to 1 pg (Figure 2A). We observed a linear dynamic range for all tested RNA concentrations. The amplification efficiencies were between 90 and 94% for all four cell lines (Figure 2A). Next, we tested our method on fluorescence-activated cell sorted (FACS) cells, ranging from 128 to single cells (Figure 2B). As for the extracted total RNA data, we observed a linear relationship between cDNA levels and cell numbers. The amplification efficiencies were between 96 and 104% (Figure 2B).

To test whether the added SYBR Green I affected the amplified transcriptome integrity, we compared preamplified cDNA with and without SYBR Green I. The preamplified cDNA was purified using magnetic beads and then evaluated by comparing their size distribution (Figure S1). Addition of SYBR Green I showed no effect on size distribution. Instead, surprisingly, the addition of SYBR Green I generated a slightly higher preamplification yield.

### 3.2. Individual Sarcoma Cells Reveal Heterogeneity in Total Polyadenylated Transcriptome Levels

**Figure 3:**
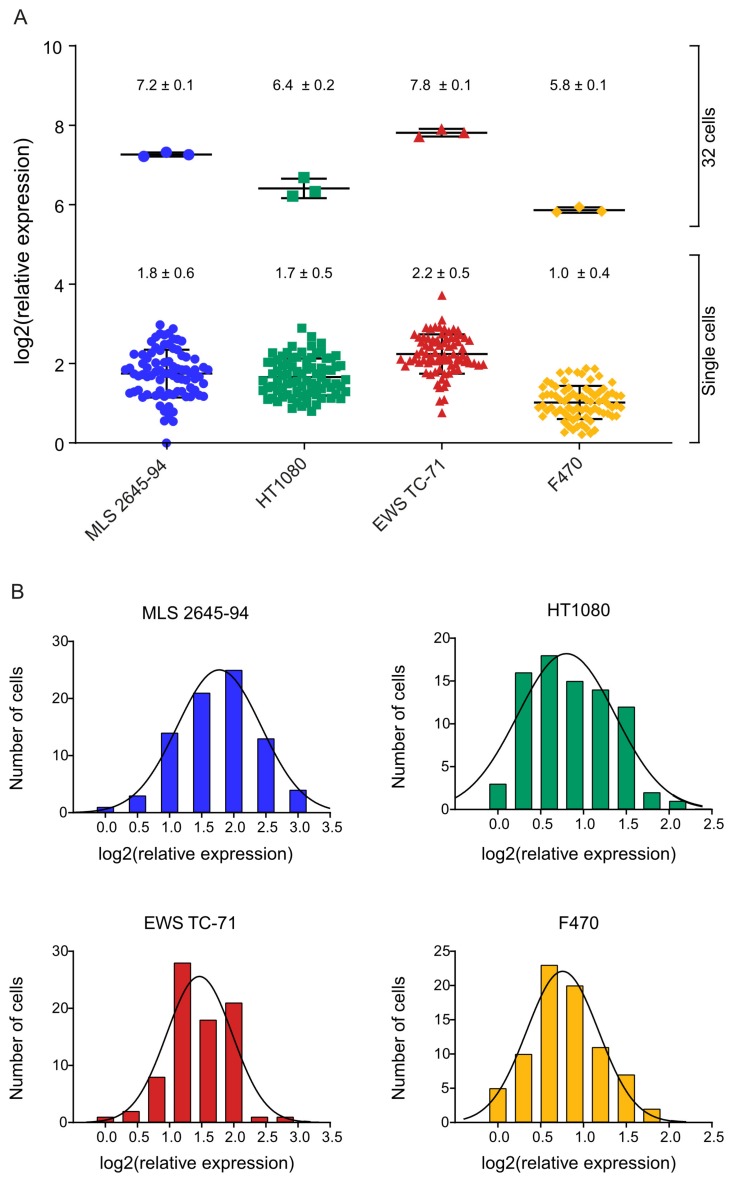
Cell heterogeneity in total polyadenylated RNA levels. (**A**) Total polyadenylated RNA levels in single cells and 32 cells from myxoid liposarcoma (MLS) 2645-94, HT1080, Ewing sarcoma (EWS) TC-71, and F470, expressed as relative quantities normalized to the mean expression of all F470 cells. Mean ± SD is indicated, *n* = 78–81 (1 cell), *n* = 3 (32 cells). (**B**) Histograms of total polyadenylated RNA levels among single cells from MLS 2645-94, HT1080, EWS TC-71, and F470. The solid line indicates the Gaussian curve fit. *n* = 78–81.

Sarcoma includes many entities with specific cellular phenotypes and unique genotypes, all with mesenchymal origin. To determine the heterogeneity in polyadenylated transcriptome levels in sarcomas, we analyzed 80–81 single cells of three representative cell lines (MLS 2645-94, HT1080, and EWS TC-71). The only known mutation in MLS 2645-94 is the fusion oncogene *FUS-DDIT3* [26]. HT1080 has reported mutations in *NRAS*, *RAC* [27], and *IDH1* [28], while EWS TC-71 harbors the fusion oncogene *EWSR1-FLI1* and mutations in *CDKN2A* and *TP53* [27]. For comparison, we also analyzed 80 individual fibroblasts (F470). Comparisons of amplification and melting curves between single cells and cell-free controls, i.e., reverse transcription negatives, showed that positive samples could be identified and separated from negative samples (Figure S2). Two out of 322 analyzed wells with sorted cells were interpreted as negative. Bulk and single-cell data demonstrated that the relative expression of polyadenylated RNA significantly varied between the different cell lines, where the EWS TC-71 cell line showed the highest expression, whereas the F470 cells showed the lowest (Figure 3A and Table S1). Also, a heterogeneity in polyadenylated transcriptome levels among the single cells within each cell line was observed, displaying log-normal distribution features (Figure 3B). The MLS 2645-94 cell line showed the highest variability with a 7.9-fold difference between the lowest expressing and highest expressing cell, while the fibroblasts showed the lowest variability with a 3.5-fold difference.

## 4. Discussion

We developed a method to quantify the amount of polyadenylated RNA in single cells, which can be used to profile global transcript differences among cell types as well as to monitor the effects of intrinsic and extrinsic factors. The protocol is simple and fast to perform without the need for sequencing. However, the approach can easily be combined with RNA sequencing using the Smart-seq2 protocol as it utilizes the same reverse transcription protocol. In a similar manner, our method can also be combined with targeted gene expression analysis, such as qPCR [25]. In this way, our approach can be useful both as an independent assay and as a readout when also profiling specific genes. Current methods to quantify the total RNA level in single cells include the use of RNA spike-in controls, such as External RNA Controls Consortium (ERCC) [29]. However, several challenges regarding the use of ERCC spike-ins have been observed, such as differences in technical effects between spike-in molecules and intrinsic genes [30]. Compared to our method, the spike-ins will only give an indirect quantification. For solely normalization purposes in regard to certain analyses, there are also computational methods that attempt to take the transcriptome size into account [31,32]. Potentially, our method can also be used to improve the normalization of sequencing and qPCR data, but this needs to be further investigated.

For efficient transcriptomic sequencing, rRNA needs to be avoided since total RNA mainly consists of rRNA [33]. Our protocol relies on oligo-dT priming in reverse transcription to select for polyadenylated RNA. This method captures most mRNA, even though some gene groups, such as histones, lack poly-A tails and will be excluded, but it will also capture many long non-coding RNAs that are polyadenylated [34]. For applications where inclusion of more RNA species is desirable, a few protocols have been developed at the single-cell level that relies on other methods to remove or avoid rRNA not solely based on oligo-dT priming [35,36,37]. Some of these can potentially be used for total RNA assessment in single cells.

One potential issue with our approach is that SYBR Green I can inhibit the PCR reaction in a concentration-dependent manner, as previously shown [38]. However, our results showed no PCR inhibition, rather the opposite, and the cDNA integrity remained intact. Further studies are needed to determine the underlying mechanisms behind the potential positive effect of adding SYBR Green I in the preamplification reaction. Our data demonstrated lower PCR efficiencies for the RNA dilutions compared to the sorted cells (90–94% compared to 96–104%, respectively). One explanation is that the RNA dilution is somewhat biased due to technical issues, such as RNA adsorption to the reaction well [39]. Amplification of cell-free control samples showed the formation of non-specific PCR products, but our single-cell analysis displayed a clear separation between single cells and cell-free controls, based on the amplification curves. Based on amplification and melting curves, we could disregard 2 out of 322 samples as empty wells or wells containing only apoptotic/necrotic cells without any mRNA. In some cell-free controls, the melting curve shapes were similar to samples with cells. This was most likely not due to sample-to-sample contamination since no amplification was detected in any qPCR negatives. Instead, cDNA was generated from nucleic acids in the reverse transcription step [40]. Most reverse transcriptases and DNA polymerases contain nucleic acid residues from the enzyme production that is amplifiable [41]. However, in our data, this background noise was not relevant, since the amount of preamplified cDNA in cell-well negatives was several times lower compared to the amount of cDNA in single cells. Further investigations are needed to determine the true origin of preamplified PCR products in reverse transcription negatives.

When comparing the total polyadenylated RNA expression between the analyzed cell lines we observed that, for both single cells and 32 cells, normal fibroblasts (F470) showed the lowest total polyadenylated RNA levels, while the EWS cell line TC-71 showed the highest (Figure 3A and Table S1). This may be correlated to the proliferation rate of the cells since the fibroblasts proliferate slowly, while TC-71 is the most fast-growing cells of the remaining three cell lines. In addition to the growth rate, the tumor cell origin and driver mutations may also affect the total polyadenylated RNA level. EWS TC-71 and MLS 2645-94 both carry specific fusion oncoproteins (EWSR1-FLI1 and FUS-DDIT3, respectively) that are known to interact with the SWI/SNF chromatin remodeling complex [42], which may affect transcriptional control at the global level. The HT1080 cell line carries no fusion oncogenes, but a number of mutations in other genes that may influence the transcriptome in a different way compared to the other cell lines. However, further studies are needed to determine the effects of specific mutations on the total polyadenylated RNA levels. We identified log-normality when studying the distribution of the total polyadenylated RNA expression levels among the single cells for all four cell types (Figure 3B). This is in agreement with previous studies of individual transcripts that have shown gamma and/or log-normal distribution features [43,44], which is in line with transcriptional bursting [45]. Comparing the expression values between single cells, we observed a 3.5 to the 7.9-fold difference between the lowest expressing and highest expressing cells in the respective cell line. Part of this variation can be explained by the fact that cells are in different cell-cycle phases. Recent studies have shown that the polyadenylated RNA level varies more than 10 times throughout the cell cycle when normalizing read counts to ERCC spike-in reads [19]. To control for cell cycle effects, cells can be synchronized or collected in the respective cell cycle phase [18,19]. The latter is preferred since cell synchronization may cause cell stress and abnormal gene expression profiles [18,46,47]. Comparison of the cell lines indicated a correlation between the variability between the individual cells and the average total polyadenylated RNA expression, where the largest variations were observed for the cell lines with the highest RNA levels. It will be interesting to assess the total amount of mRNA in various healthy and pathological cell types in combination with controlled perturbations in intrinsic and extrinsic factors to determine the role of global transcriptional regulation.

## Supplementary Materials

The following are available online at https://www.mdpi.com/2073-4409/9/3/759/s1, Figure S1: Size distribution assessment of preamplified cDNA, Figure S2: Preamplification and melting curves, Table S1: Pairwise statistical comparisons of relative expression levels between cell lines.
