# Supplementary material for: Total mRNA Quantification in Single Cells: Sarcoma Cell Heterogeneity
Source: Cells. 2020 Mar 19;9(3):759. doi: 10.3390/cells9030759 (PMC7140709; doi:10.3390/cells9030759)
Supplement: Supplementary file 1 [file cells-09-00759-s001.pdf]

Supplementary Material

Article

Total mRNA Quantification in Single Cells: Sarcoma Cell Heterogeneity

Emma Jonasson [1], Lisa Andersson [1], Soheila Dolatabadi [1], Salim Ghannoum [1], Pierre Åman [1] and Anders Ståhlberg [1,2,3,*]

[1] Sahlgrenska Cancer Center, Department of Laboratory Medicine, Institute of Biomedicine, Sahlgrenska Academy at University of Gothenburg, SE-405 30, Gothenburg, Sweden; emma.jonasson@gu.se (E.J.); lisa.andersson.3@gu.se (L.A.); soheila.dolatabadi@gu.se (S.D.); salim.ghannoum@medisin.uio.no (S.G.); pierre.aman@gu.se (P.Å.)

[2] Department of Clinical Genetics and Genomics, Sahlgrenska University Hospital, SE-405 30, Gothenburg, Sweden

[3] Wallenberg Centre for Molecular and Translational Medicine, University of Gothenburg, SE-405 30, Gothenburg, Sweden

* Correspondence: anders.stahlberg@gu.se; Tel.: +46-31-7866735

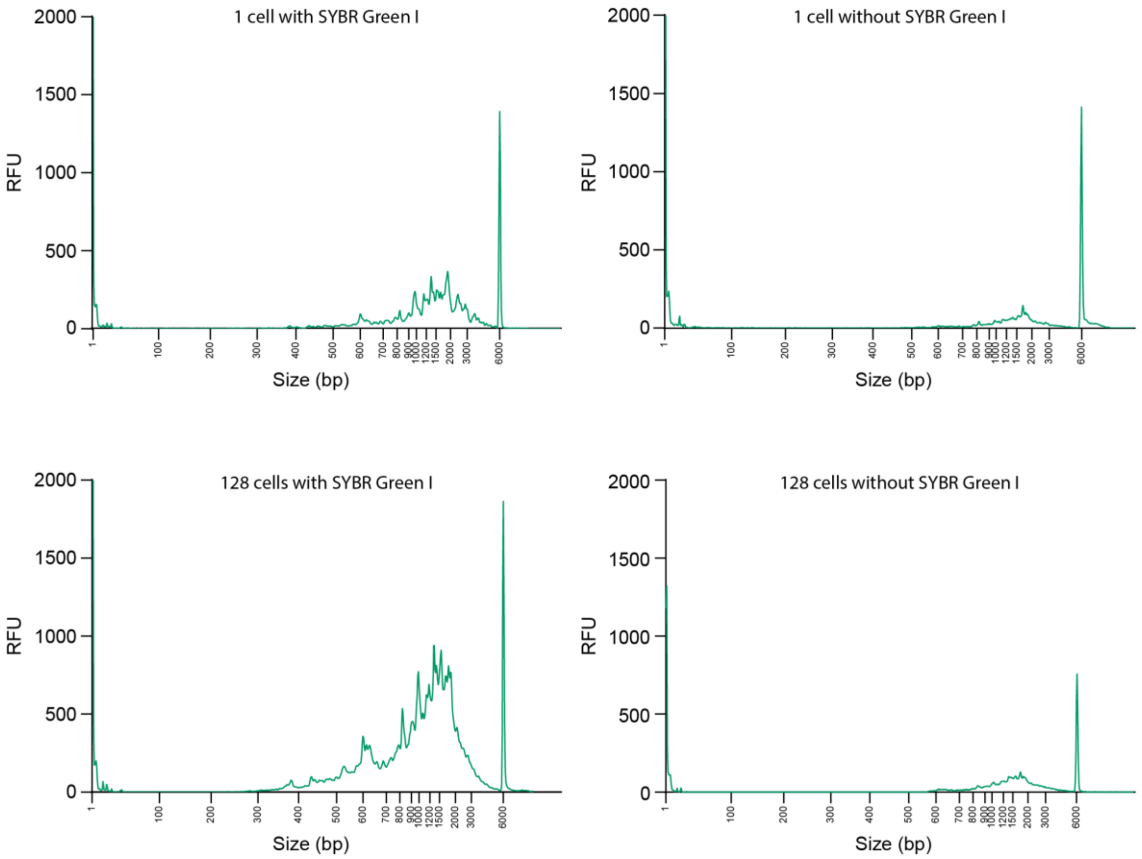

Figure S1. Size distribution assessment of preamplified cDNA. Size distribution analysis using Fragment Analyzer analysis of purified cDNA, preamplified for a limited number of cycles with and without SYBR Green I added to the reaction. Representative electropherograms for 128 cells and single cells are shown. RFU, Relative fluorescence unit.

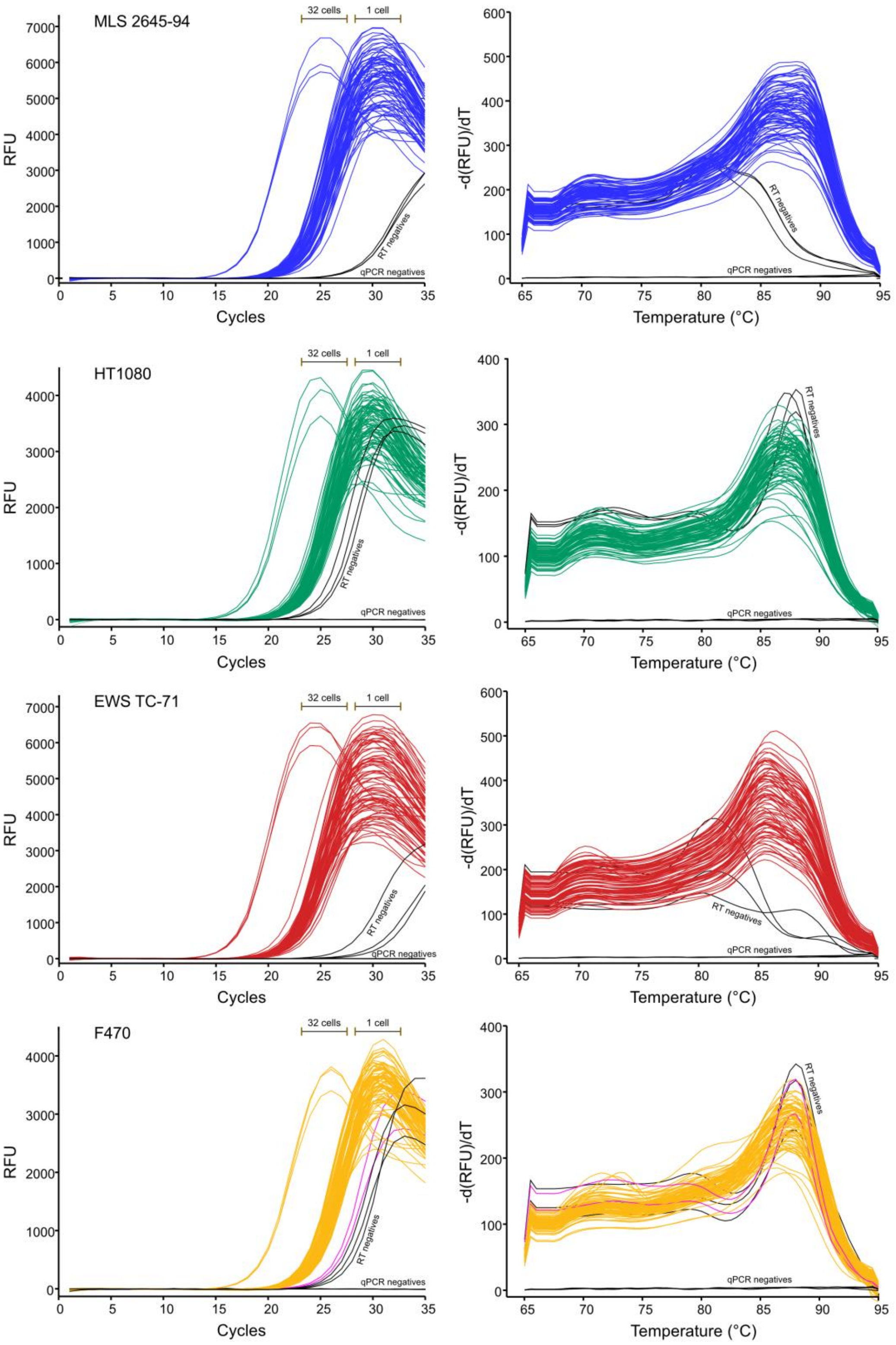

Figure S2. Preamplification and melting curves. (**A**) Preamplification curves of cDNA generated from myxoid liposarcoma (MLS) 2645-94, HT1080, Ewing sarcoma (EWS) TC-71 and F470. Single cells (n = 79–81), 32 cells (n = 3), cell-free negatives, *i.e.* reverse transcription negatives and qPCR negatives (n = 3 each). The two samples in magenta were interpreted as empty wells or apoptotic/necrotic cells. (**B**) Melting curves of preamplified cDNA from MLS 2645-94, HT1080, EWS TC-71 and F470. Single cells (n = 79–81), 32 cells (n = 3), cell-free negatives, *i.e.* reverse transcription negatives and qPCR negatives (n = 3 each). The two samples in magenta were interpreted as empty wells or apoptotic/necrotic cells. RFU, Relative fluorescence unit; T, Temperature.

Table S1. Pairwise statistical comparisons of relative expression levels between cell lines. Statistical tests were performed with Tukey's multiple comparison test.

Single cells	Mean difference	Adjusted P value
MLS 2645-94 vs. HT1080	0.09	0.67
MLS 2645-94 vs. EWS TC-71	−0.49	<0.01
MLS 2645-94 vs. F470	0.73	<0.01
HT1080 vs. EWS TC-71	−0.58	<0.01
HT1080 vs. F470	0.64	<0.01
EWS TC-71 vs. F470	1.22	<0.01
**32 cells**	**Mean difference**	**Adjusted P value**
MLS 2645-94 vs. HT1080	0.85	<0.01
MLS 2645-94 vs. EWS TC-71	−0.55	<0.01
MLS 2645-94 vs. F470	1.40	<0.01
HT1080 vs. EWS TC-71	−1.40	<0.01
HT1080 vs. F470	0.54	<0.01
EWS TC-71 vs. F470	1.95	<0.01
